# Evaluation and breeding application of six brown planthopper resistance genes in rice maintainer line Jin 23B

**DOI:** 10.1186/s12284-018-0215-4

**Published:** 2018-04-11

**Authors:** Haichao Jiang, Jie Hu, Zhi Li, Jia Liu, Guanjun Gao, Qinglu Zhang, Jinghua Xiao, Yuqing He

**Affiliations:** National Key Laboratory of Crop Genetic Improvement, National Center of Plant Gene Research (Wuhan), and National Center of Crop Molecular Breeding, Huazhong Agricultural University, Wuhan, 430070 China

**Keywords:** *Oryza sativa* L, Brown planthopper resistance, Marker-assisted selection (MAS), Gene introgression

## Abstract

**Background:**

The brown planthopper (BPH), an insect species that feeds on rice plants (*Oryza sativa* L.), is one of the most destructive insect pests of rice. BPH can be controlled with chemical pesticides, but they are expensive in terms of the cost and environmental hazards. Hence, utilization of resistance genes in resistant varieties is considered as a more economical and eco-friendly effective method for control.

**Results:**

In this study, six dominant BPH-resistance genes (*Bph3*, *Bph14*, *Bph15*, *Bph18*, *Bph20* and *Bph21*) were introduced into an elite *indica* rice cultivar Jin 23B using the marker-assisted selection (MAS) method of breeding. One line combining *Bph14* and *Bph15* and six single gene introgression lines were used to evaluate the gene effects based on three parameters: seedling and tillering resistance of the rice genotypes, honeydew weight, and BPH survival rate. Among all improved lines, combination of *Bph14* and *Bph15* had the largest effect in conferring resistance to BPH. Bioassays showed that the order of the gene effects was *Bph14*/*Bph15* > *Bph15* ≥ *Bph14* ≥ *Bph20* ≥ *Bph21* ≥ *Bph3* > *Bph18* > none at the rice seedling stage. The pyramided or single-gene introgression lines showed enhanced resistance relative to the control. Furthermore, field trial data demonstrated that yields of improved Jin 23B lines were similar to the control under BPH-free field conditions.

**Conclusions:**

Each of the BPH resistance genes reduced BPH growth and development, and was effective at both the seedling and tillering growth stages. These lines can be used in rice hybrid and production in China.

## Background

Brown planthopper (*Nilaparvata lugens* Stål, BPH), is one of the most destructive insect pests of tropical and temperate rice (*O. sativa* L.) in Asia. BPH extracts phloem saps from host plants using its stylet-type mouthparts, resulting in severe and damaging symptom known as ‘hopper-burn’ (Watanabe and Kitagawa 2000). BPH also transmits viruses (Cha et al. 2008), such as grassy stunt virus and rugged stunt virus to rice plants, causing further yield losses in Asian countries, including China, Japan, Korea and Vietnam (Brar et al. 2010). In rice production, BPH is managed mainly by using insecticides that are expensive in terms of cost and environmental hazards. Therefore, utilization of BPH resistance genes in developing resistant varieties is considered as the most economical and eco-friendly approach to control this pest (Tanaka et al. 2000).

More than 32 BPH resistance loci have been identified in *indica* rice cultivars and wild rice species (Ali and Chowdhury 2014; Wang et al. 2015). Several of these resistance genes have been assigned to rice chromosomes 2, 3, 4, 6, and 12 (Jena and Kim 2010) and at least ten genes have been fine-mapped to genomic region of less than 200 kb (Hu et al. 2016). Most of the resistance alleles are dominant, but a few are documented as recessive (*bph4*, *bph5*, *bph7*, *bph8*, *bph19*,*bph25* and *bph29*). Seven BPH-resistance genes *Bph14*, *Bph26*, *Bph17*, *bph29*, *Bph9*, *Bph18* and *Bph32* have been cloned by map-based cloning (Du et al. 2009; Tamura et al. 2014; Liu et al. 2015; Wang et al. 2015; Zhao et al. 2016; Ji et al. 2016; Ren et al. 2016). Among the resistance loci, the broad-spectrum resistance gene *Bph3*, identified in cultivars Rathu Heenati and PTB33, was mapped on the short arm of chromosome 6 where it was flanked by SSR markers RM589 and RM588 (Jairin et al. 2007a). *Bph14* and *Bph15* were previously mapped in introgression line B5 (derived from *O. officinalis*), with *QBph1* (renamed as *Bph14*) on the long arm of chromosome 3 explaining 26.4% of the variation in BPH response and *Qbph2* (renamed as *Bph15*) on the short arm of chromosome 4 explaining 14.3% of the variation (Huang et al. 2001). *Bph14*, encoding a coiled-coil, nucleotide-binding, and leucine-rich repeat protein was cloned using a map-based cloning approach (Du et al. 2009). *Bph15* was fine mapped to an interval of approximately 50 kb between map positions 6,883,264 and 6,934,420 (Yang et al. 2004). The *Bph18* gene, originally derived from *O. australiensis*, was mapped in *indica* introgression line IR65482–7–216-1-2, shown to be on the long arm of chromosome 12, and explained 47% of the variation in BPH response (Jena et al. 2006). Map-based cloning and complementation test revealed that *Bph18* encodes a CC-NBS-NBS-LRR protein and is a functionally different allele of *Bph26* (Ji et al. 2016). *Bph20* and *Bph21* in introgression line IR71033–121-15 were derived from *O. minuta* (Accession number 101141), with *Bph20* located to a 193.4 kb region on the short arm of chromosome 4, and with *Bph21* located to a 194 kb region on the long arm of chromosome 12 (Rahman et al. 2009).

Near-isogenic lines (NILs) and gene combination lines carrying one or several resistance genes have been developed (Sharma et al. 2004; Hu et al. 2012, 2013) and in an effective manner to facilitate the use of the resistance genes or of quantitative trait loci (QTLs) for future rice improvement program. *Bph1*, *Bph3* and *Bph4* have already been successfully introduced into some elite cultivars using MAS and backcross breeding programs at the International Rice Research Institute (IRRI) in the Philippines (Khush and Brar 1991; Jena and Kim 2010). Qiu et al. (2011) used MAS to pyramid *Bph6* and *Bph12* into a *japonica* line and an *indica* line. Results of BPH bioassays indicated that the pyramided line showed higher resistance than the *Bph6-* or *Bph12*-single introgression line. Hu et al. (2012) incorporated *Bph14* and *Bph15* genes into Minghui 63 and its hybrid Shanyou 63, and showed that hybrids containing a single BPH resistance gene had enhanced resistance compared to conventional hybrids that lack of resistance gene. Lines combining two genes were more resistant than the respective single gene lines. Hu et al. (2013) incorporated *Bph14*, *Bph15* and *Bph18* into cv. 9311 and its hybrids GZ63-4S/9311 and Y58S/9311. Test of BPH response showed that lines with three gene combined were more resistant than lines with two genes or lines with single genes.

The three-line hybrid breeding system is an important method for producing commercial rice hybrids in China and elsewhere. The system involves a cytoplasmic male sterile (CMS) line (A), a fertile maintainer line (B), and a restorer line. The A line is used as a female parent and the restorer line is used as male parent for hybrid seed production. The B line is near-isogenic to the A-line except for its pollen fertility. The B line has viable pollen grains and normal seed setting, so it can pollinate the A line, while the F_1_ plants from this pollination are male sterile, again. In this way, the male sterility of the A line is maintained, and the A line is reproduced for further use or commercial use in a large scale. Jin 23B is a maintainer line of male-sterile Jin 23A, whereas Jin 23A is an elite *indica* cytoplasmic male sterile (CMS) line widely used to produce three-line hybrids over the last 20 years in China. More than 100 different hybrids are derived from Jin 23A (http://www.ricedata.cn/), and in 2008 and 2009, Jin 23A-derived hybrid varieties are grown on about 1.7 million ha of rice in China with the highest ranking (Shi et al. 2012). However, these hybrids and their parents have become increasingly susceptible to blast and BPH due to absence of resistance genes and narrow genetic diversity. Blast resistance was recently improved by the introduction of genes *Pi1*, *Pi2* and *D12*, into Jin 23B by marker assisted backcross breeding (Jiang et al. 2012). To similarly improve BPH resistance, we introduced BPH resistance genes *Bph3*, *Bph14*, *Bph15*, *Bph18*, *Bph20* and *Bph21* into Jin 23B with comprehensive evaluation. These lines will contribute to ensuring the security of rice production in China.

## Methods

### Plant materials and insects

PTB33 (carrying *Bph3*, abbreviated as 3), served as the donor of *Bph3*. B5, an introgression line derived from *O. officinalis* (Huang et al. 2001), was the donor of resistance genes *Bph14* and *Bph15* (abbreviated as 14 and 15). IR65482–7–216-1-2 (carrying *Bph18*, abbreviated as 18), an introgression line from *O. australiensis* (Acc. No. 10082), served as the donor of *Bph18*. IR71033–121-15 (carrying *Bph20* and *Bph21*, abbreviated as 20 and 21), served as the donor of *Bph20* and *Bph21*. B5 was obtained from College of Life Sciences, Wuhan University, China. IR65482–7–216-1-2, IR71033–121-15 and PTB33 were obtained from the Plant Breeding, Genetics, and Biotechnology Division of the International Rice Research Institute (IRRI), Los Baños, Philippines. Jin 23B, an elite maintainer *indica* rice obtained from Hunan Hybrid Rice Research Center, Changsha, China, was used as the recurrent parent. PTB33 and Taichung Native 1 (TN1; no resistance) were used as checks in evaluation of BPH resistance.

The BPH used for infestation were collected from rice fields at Wuhan, in 2012, and were maintained in a cage by continuous rearing on the susceptible variety TN1, at the glasshouse facility at Huazhong Agricultural University. Before use in experiments, one- to five-instar BPH nymphs were prepared and maintained on TN1.

### Molecular markers used for MAS

SSR markers RM589 and RM19324 (F: GAGGTTGTTTGGATGGATAGATGG and R: AATCCCGTCCTAGAGTTCTTCTACC) were used to detect the presence of *Bph3* (Jairin et al. 2007a). The co-segregating insertion/deletion (InDel) marker 76–2 was used for *Bph14* identification (Du et al. 2009). The *Bph15* gene was identified using simple sequence repeat (SSR) marker RM261 and InDel marker 15–6 (Huang et al. 2001; Yang et al. 2004; Hu et al. 2012). SSR markers RM3331 (F: CCTCCTCCATGAGCTAATGC and R: AGGAGGAGCGGATTTCTCTC), RM28427 (F: CTGTGAGAAGGTTGAGACTTGAAAGG and R: GCAAATGCTCAAGTGAAGTTGG) and RM28561 (F: CTTCAAGACTGGCCCAATATTACTGC and R: TGACTGAAGCCTTCTTCACTTGC), were used to detect the presence of *Bph18*. SSR marker RM16553 (F: CATAGCCACTTATCGTTGTTACGC and R: TGTCCATCTATGACTGTCCACTACG) and Indel marker HJ34 (F: GCCGAATGGTAAGAAGAG and R: GCGAGTTAACCAATGCTTGG) were used to detected the presence of the *Bph20* gene. SSR marker RM28561 (F: CTTCAAGACTGGCCCAATATTACTGC and R: TGACTGAAGCCTTCTTCACTTGC) and PCR-based marker B121 were used to detected the presence of the *Bph21* gene (Rahman et al. 2009). SSR analysis was carried out according to the procedures described by McCouch et al. (2002). The *Bph3*, *Bph14*, *Bph15*, *Bph18*, *Bph20*, and *Bph21* genes were introduced into Jin 23B following a MAS-based backcross breeding strategy. Finally, six single gene introgression lines containing *Bph3*, *Bph14*, *Bph15*, *Bph18*, *Bph20* and *Bph21* and a pyramided line containing *Bph14* and *Bph15* were obtained. The selected lines maintained the excellent quality and yield potential of Jin 23B. A total of seven homozygous gene combinations (designated as 14/15, 3, 14, 15, 18, 20 and 21) were represented.

The whole-genome single nucleotide polymorphism (SNP) array RICE6K was used for genetic background profiling of the introgression lines (Yu et al. 2014). Genomic DNA from each line was extracted from 10 dry seeds. DNA amplification, fragmentation, chip hybridization, single base extension, staining and scanning were conducted by the Life Science and Technology Center, China National Seed Group Co., LTD (Wuhan, China).

### Evaluation of BPH response in seedlings and tillering stage of plants

An Experiment was conducted by using a modified bulk method under sophisticated greenhouse condition followed Pathak et al. (1969). PTB33 and TN1 were included as controls in all experiments. Lines were sown in plastic trays (60 cm length × 40 cm width × 10 cm height) with 12 plants per row, 11 rows per tray. When seedlings at 10 days post sowing (three-leaf stage) were infested with second and third instar nymphs (twelve nymphs per seedling). Each experiment was conducted in a randomized complete block design with six replications. Plant responses were recorded seven days after infestation (DAI) when all seedlings of TN1 were almost dead. BPH resistance was evaluated with the six-scale standard of scoring system, described by Huang et al. (2001): 0 = no damage; 1 = very slight damage; 3 = first and second leaves partially yellowing; 5 = pronounced yellowing and stunting; 7 = mostly wilting, the plant still alive; 9 = the plant completely wilted or died, in which higher scores indicate greater susceptibility to BPH.

The recurrent parent, backcross lines, resistant check and susceptible check (TN1) seeds were sown in the field at Wuhan for plant BPH response assessments. Fifteen days later two plants of each line and checks were transplanted into a plastic pot (16.5 cm of bottom diameter × 22.5 cm of top diameter × 19 cm of height) containing pulverized soil in ten replications. All the plants were grown in a cage at the glasshouse facility. At the tillering stage (30 days post transplanting) the plants were infested with second and third instar nymph at a density of 300 nymphs per plant. When the susceptible check was almost dead (approximately 17 days after infestation) all plants were assessed and scored as resistant (R), moderately resistant (MR), moderately susceptible (MS) and highly susceptible (S) (Suh et al. 2011). Scoring occurred a second time about 25 days post infestation.

### Quantification of honeydew excretion from adult BPH females

Honeydew collection was accomplished under aseptic condition under greenhouse followed by Pathak et al. (1982) with minor modifications. Seven improved rice families with BPH resistance gene *Bph3*, *Bph14*, *Bph15*, *Bph18*, *Bph20*, *Bph21* and *Bph14*/*Bph15* and the control Jin 23B and PTB33 were planted in plastic pots. 50 days after transplanting, five-instar BPH female nymph (previously starved for 2 h) were inoculated with a suitable parafilm sheet (3.5 × 4.5 cm) (Incubation rate = two females per sheet) and subjected to the improved lines with suitable check. After 24 h of feeding, insects were removed from the sheet, and honeydew in each sheet were weighted using a 0.1 mg sensitivity balance. An experiment was repeated over four replications.

### BPH survival on rice plants

BPH survival measurements were made on the backcross lines and suitable checks following the method of Sebastian et al. (1996). Individual seedlings were grown on a rice liquid culture medium in 500 ml volumetric flasks. Ten insects with second-instar nymph were subsequently put into each volumetric flask, and survivability of BPHs in each flask were recorded in every 2 days of interval for 10 days. The experiment was carried out in greenhouse, and the temperature was maintained at 25–28 °C. BPH survival percentage was calculated as follows. BPH survival percentage = Number of surviving nymph divided by the total number of released nymph. This measurement was repeated for six times.

### Pollen fertility of improved lines

Fertility assessments of the backcross lines were made using six to ten selected lines for each genotype. Crosses were made to the male sterile Jin 23A, and the F_1_ plants were used as female parents in crosses with the corresponding improved lines. Six spikelets per line of each F_1_ and BC_1_F_1_ individual were sampled for assessment of pollen fertility using 1% I2-KI staining (Virmani et al. 1997). The fertility rate of each line was scored as completely sterile (0%), partially sterile (1–30%), partially fertile (> 30–60%) and fertile (> 60%) (Govinda Raj and Virmani 1988).

### Evaluation of agronomic traits in the field

Agronomic assessments of the seven backcross lines were carried under natural field conditions, seven homozygous rice families and the control (Jin 23B) were planted in randomized complete block design in Wuhan, China in the autumn of 2013. A trial was performed with six replications, each plot consisted of three rows and each row consisted of 12 plants at a planting density of 17 × 27 cm (17 cm plant to plant and 27 cm row to row distance). Seven plants from central row of each plot were subjected to agronomic trait measurements, including days to heading (DTH) and plant height (PH). After the maturity of rice, the seven plants from central row were harvested individually for panicle number (PN), number of grains per panicle (NGP), spikelet fertility (SF), weight of 1000-grains (GW) and yield per plant (YD) measurement. Filed management followed normal agricultural practices with the nitrogen, phosphorus, potassium fertilizer of 180, 65, and 65 kg/hm^2^ respectively.

### Data analysis

The available RM marker series were searched in the rice genomic database (www.gramene.org). InDel markers were designed based on the references genome of Nipponbare and 9311 reference sequences. Date analysis was performed using one-way ANOVA and Tukey’s tests in Microsoft Excel 2003 or SPSS 17.0.

## Results

### Development of introgressed and pyramided lines of Jin 23B using marker assisted selection

**Fig. 1:**
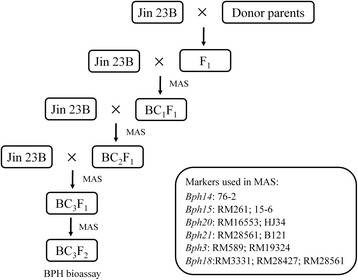
Scheme of marker-assisted backcross breeding used in the current study. Donor parents included 4 cultivars: PTB33 (*Bph3*), B5 (*Bph14*/*Bph15*), IR65482–7–216-1-2 (*Bph18*) and IR71033–121-15 (*Bph20*/*Bph21*). MAS: marker-assisted selection

BPH resistance genes were introgressed from donor parents into Jin 23B following a recurrent backcrossing procedure combined with MAS (Fig. 1). F_1_ progenies obtained from crosses between Jin 23B and each donor, namely PTB33 (carrying *Bph3*), B5 (carrying *Bph14*/*Bph15*), IR65482–7–216-1-2 (carrying *Bph18*), IR71033–121-15 (carrying *Bph20*/*Bph21*). In each BC generation, positive individuals were identified using corresponding markers, and then used for further backcrossing with the recurrent parent Jin 23B. In the BC_1_F_1_ population, 19, 27, 21, 22, 24, 23 and 19 individuals were identified for genes *Bph3*, *Bph14*, *Bph15*, *Bph18*, *Bph20*, *Bph21* and *Bph14*/*Bph15* respectively. In the BC_2_F_1_ population, 18, 25, 29, 24, 22, 25 and 18 individuals were identified for gene *Bph3*, *Bph14*, *Bph15*, *Bph18*, *Bph20*, *Bph21* and *Bph14*/*Bph15*. These plants were subsequently backcrossed to Jin 23B by mixing pollen grains. After three generation of backcrossing, homozygous BC_3_F_2_ plants were selected (Fig. 1). With dual selection (MAS + BPH bioassay) and further field selection we obtained sets of homozygous lines for *Bph3*, *Bph14*, *Bph15*, *Bph18*, *Bph20*, *Bph21* and *Bph14/15*, which were renamed as Jin 23B (3) (16 lines), Jin 23B (14) (28 lines), Jin 23B (15) (26 lines), Jin 23B (18) (23 lines), Jin 23B (20) (16 lines), Jin 23B (21) (25 lines) and Jin 23B (14/15) (15 lines). Following self-pollinating and dual selection, Jin 23B improved lines with enhanced BPH resistance and high yield were developed.

### Genetic background examination of the introgressed lines and pyramided line

**Fig. 2:**
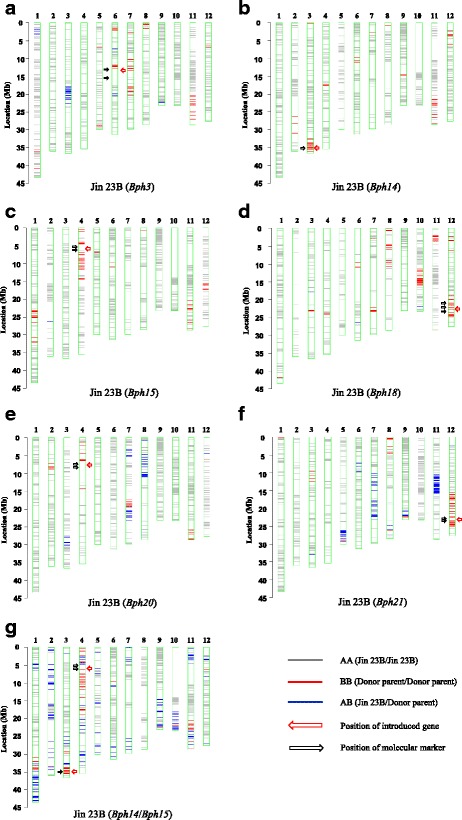
Haplotype maps of genetic background profiling of improved Jin 23B using the RICE6K array. (**a**) Jin 23B (*Bph*3), (**b**) Jin 23B (*Bph*14), (**c**) Jin 23B (*Bph*15), (**d**) Jin 23B (*Bph*18), (**e**) Jin 23B (*Bph*20), (**f**) Jin 23B (*Bph*21), (**g**) Jin 23B (*Bph*14/ *Bph*15). Donor parents included 4 cultivars: PTB33 (*Bph3*), B5 (*Bph14*/*Bph15*), IR65482–7–216-1-2 (*Bph18*) and IR71033–121-15 (*Bph20*/*Bph21*). Arrowheads with red color indicate the positions of introduced gene. Arrowheads with black color indicate the positions of molecular marker used in marker-assisted selection backcross breeding program

In this study, RICE6K, a whole-genome single nucleotide polymorphism (SNP) array was used to analyze the genetic background of the introgressed line and pyramided line. This array contained 1111 polymorphic SNP markers between Jin 23B and PTB33, 1040 polymorphic SNP markers between Jin 23B and B5, 682 polymorphic SNP markers between Jin 23B and IR65482–7–216-1-2, and 1050 polymorphic SNP markers between Jin 23B and IR71033–121-15. Populations developed after three backcrosses are expected to have about 87.5% recovery of the recurrent parent genotype. As shown in Fig. 2 the genetic background recoveries of the recurrent parent in Jin 23B (3), Jin 23B (14), Jin 23B (15), Jin 23B (18), Jin 23B (20), Jin 23B (21) and Jin 23B (14/15) were 91.72%, 94.13%, 92.02%, 86.95%, 90.67%, 83.43% and 77.11%, respectively, measured by percentage of the polymorphic marker ratios. Large fragments of donor chromatin were present in the positions of *Bph3* in chromosome 6, *Bph14* in chromosome 3, *Bph15* and *Bph20* in chromosome 4, and *Bph18* and *Bph21* in chromosome 12, because of foreground selection of the target gene in each generation by MAS.

### BPH responses of the introgression lines at seedling and tillering stages

**Fig. 3:**
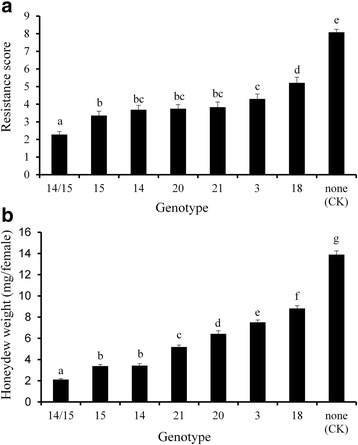
**a** The resistance scores of pyramided line and introgression lines of Jin 23B conferring to BPH at seedling stage. The resistance score was determined by the mean of score from at least 30 lines each homogenous allele combination. **b** The honeydew weight per BPH females on improved lines of Jin 23B. Number 14/15 represent Jin 23B (*Bph14*/*Bph15*), 15 represent Jin 23B (*Bph15*), 14 represent Jin 23B (*Bph14*), 20 represent Jin 23B (*Bph20*), 21 represent Jin 23B (*Bph21*), 3 represent Jin 23B (*Bph3*) and 18 represent Jin 23B (*Bph18*). none (CK): Jin 23B. The different letters above the bars are significantly different at *P* < 0.05 level

**Fig. 4:**
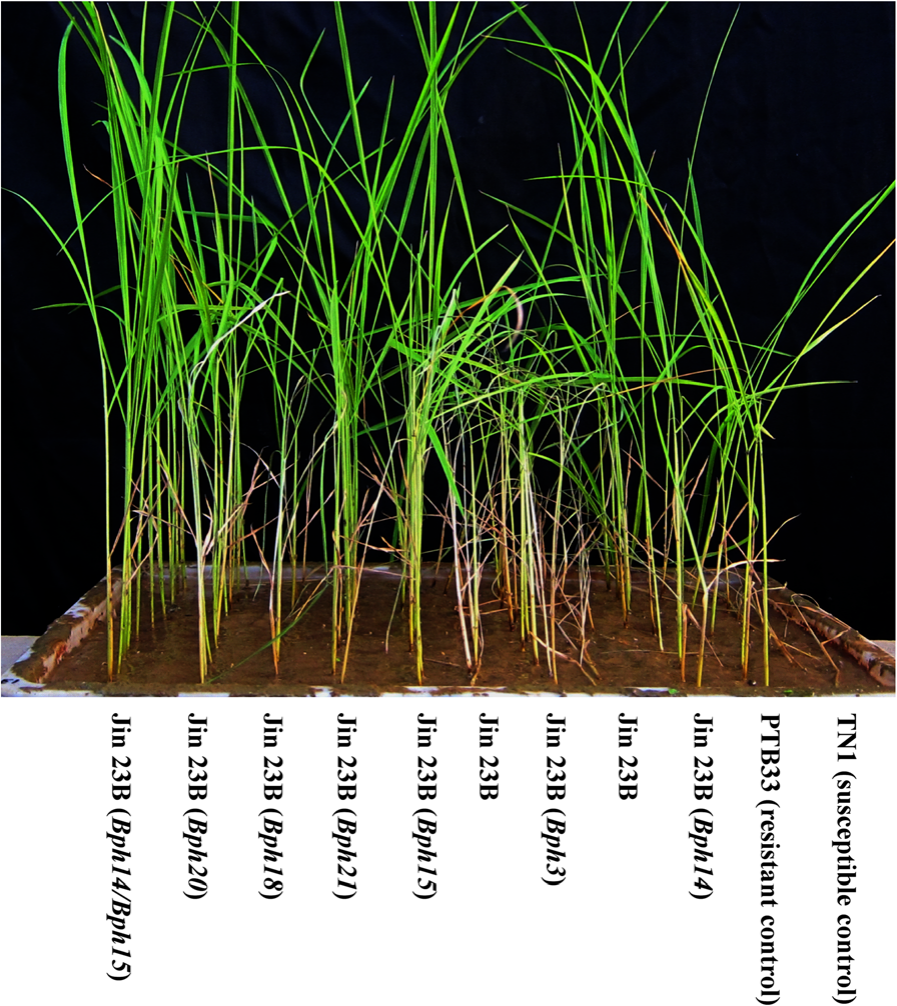
Photograph of resistance level to BPH of improved Jin 23B with resistance gene *Bph3*, *Bph14*, *Bph15*, *Bph18*, *Bph20*, *Bph21* and *Bph14*/*Bph15* at the seedling stage (the 7 days after infested with BPH). PTB33 (carrying *Bph3*) is a resistance control and TN1 is a susceptible control

Introgression lines with homozygous *Bph3*, *Bph14*, *Bph15*, *Bph18*, *Bph20* and *Bph21* were used for evaluation of BPH-resistance at both seedling and tillering stage. At the seedling stage (Figs. 3a, 4), the resistance score for Jin 23B harboring *Bph14* and *Bph15*, designated as Jin 23B (14/15), account 2.28, which conferred highest resistance rating among all the selected lines. Introgression lines containing single *Bph* genes, Jin 23B (15), Jin 23B (14), Jin 23B (20), Jin 23B (21), Jin 23B (3), and Jin 23B (18), with desirable resistance scores were found 3.36, 3.69, 3.75, 3.83, 4.3 and 5.21, respectively, and that for the donor parents PTB33 (carrying *Bph3*), B5 (carrying *Bph14*/*Bph15*), R65482–7–216-1-2 (carrying *Bph18*) and IR71033–121-15 (carrying *Bph20*/*Bph21*) the resistance scores were 3.21, 2.39, 3.86 and 2.67, respectively. In contrast Jin 23B (none) control scored 8.08 (Figs. 3a, 4). For single *Bph* gene lines, Jin 23B (15) conferred the highest seedling resistance level whereas Jin 23B (18) conferred the lowest seedling resistance level. Furthermore, the pyramided line with two resistance genes (*Bph14*/*Bph15*) showed significantly higher resistance compared to the ones with single resistance gene.

**Fig. 5:**
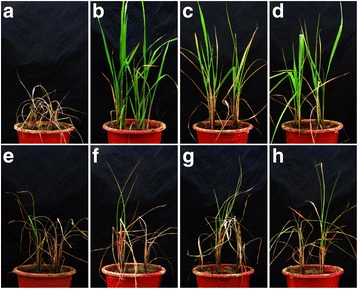
Photograph of resistance level to BPH of improved Jin 23B at the tillering stage (the 25 days after infested with BPH). Number A to H represent Jin 23B, Jin 23B (*Bph14*/*Bph15*), Jin 23B (*Bph14*), Jin 23B (*Bph15*), Jin 23B (*Bph3*), Jin 23B (*Bph18*), Jin 23B (*Bph20*), Jin 23B (*Bph21*), respectively

At the tillering stage, all the plants grown in a cage infested with BPH were scored twice to evaluate the resistance degree. Lines Jin 23B (14/15), Jin 23B (15), Jin 23B (14) were classified R to BPH, Jin 23B (20), Jin 23B (21), Jin 23B (3), Jin 23B (18) were MR to BPH, whereas Jin 23B (none) was MS to BPH at 17 days post infestation (Table 1, Fig. 5). Lines Jin 23B (14/15), Jin 23B (15), Jin 23B (14) were classified MR to BPH and Jin 23B (20), Jin 23B (21), Jin 23B (3), Jin 23B (18) were MS to BPH, whereas Jin 23B (none) was S to BPH at the 25 days after infestation (Table 1, Fig. 5). In brief, at tillering stage, Jin 23B (14/15), Jin 23B (15) and Jin 23B (14), showed R to MR to BPH from 17 DAI to 25 DAI, and Jin 23B (20), Jin 23B (21), Jin 23B (3) and Jin 23B (18) showed MR to MS to BPH from 17 DAI to 25 DAI. Whereas Jin 23B (none) showed MS to S to BPH from 17 DAI to 25 DAI.

**Table 1:** The tillering resistance of different genotype of *Bph3*, *Bph14*, *Bph15*, *Bph18*, *Bph20* and *Bph21* conferring to BPH 17 DAI and 25 DAI

	Tillering resistance (17DAI → 25DAI^b^)							
Genotypes^a^	14/15	15	14	20	21	3	18	none (CK)
Resistance level	R → MR	R → MR	R → MR	MR → MS	MR → MS	MR → MS	MR → MS	MS → S

^a^14/15: 14/15: pyramided lines containing both *Bph14* and *Bph15* genes, 15: introgression lines containing *Bph15* gene, 14: introgression lines containing *Bph14* gene, 20: introgression lines containing *Bph20* gene, 21: introgression lines containing *Bph21* gene, 3: introgression lines containing *Bph3* gene, 18: introgression lines containing *Bph18* gene, none (CK): Jin 23B

^b^DAI: days after infestation. *R* resistant, *MR* moderately resistant, *MS* moderately susceptible, *S* susceptible

In general, for pyramided and introgression lines at seedling stage, the order of the gene effect was: *Bph14*/*Bph15* > *Bph15* ≥ *Bph14* ≥ *Bph20* ≥ *Bph21* ≥ *Bph3* > *Bph18* > none (Fig. 3a). At tillering stage, the order of the gene effect was: *Bph14*/*Bph15* = *Bph15* = *Bph14* > *Bph20* = *Bph21* = *Bph3* = *Bph18* > none (Table 1, Fig. 5).

### Honeydew weight excreted from BPH

To determine the effects of resistance genes in BPH growth and development, we compared the honeydew weight excreted from BPH after feeding with improved lines (Fig. 3b). For the pyramided or introgression lines, the BPH honeydew weights were gradually increased from 2.11 mg/female on Jin 23B (14/15) to 8.82 mg/female on Jin 23B (18) (Fig. 3b). The honeydew weight for Jin 23B (15), Jin 23B (14), Jin 23B (21), Jin 23B (20), Jin 23B (3) were 3.39, 3.43, 5.19, 6.43 and 7.51 mg/female, respectively (Fig. 3b). In comparison, the negative control, Jin 23B (none), produced the highest honeydew weight of 13.89 mg/female (Fig. 3b), and the resistance control PTB33, B5, IR65482–7–216-1-2 and IR71033–121-15 produced of 3.86, 2.89, 7.12 and 3.36 mg/female. Taken together, the order of the gene effect in BPH honeydew weight was *Bph14*/*Bph15* > *Bph15* ≥ *Bph14* > *Bph21* > *Bph20* > *Bph3* > *Bph18* > none (Fig. 3b).

### BPH survival rates

**Fig. 6:**
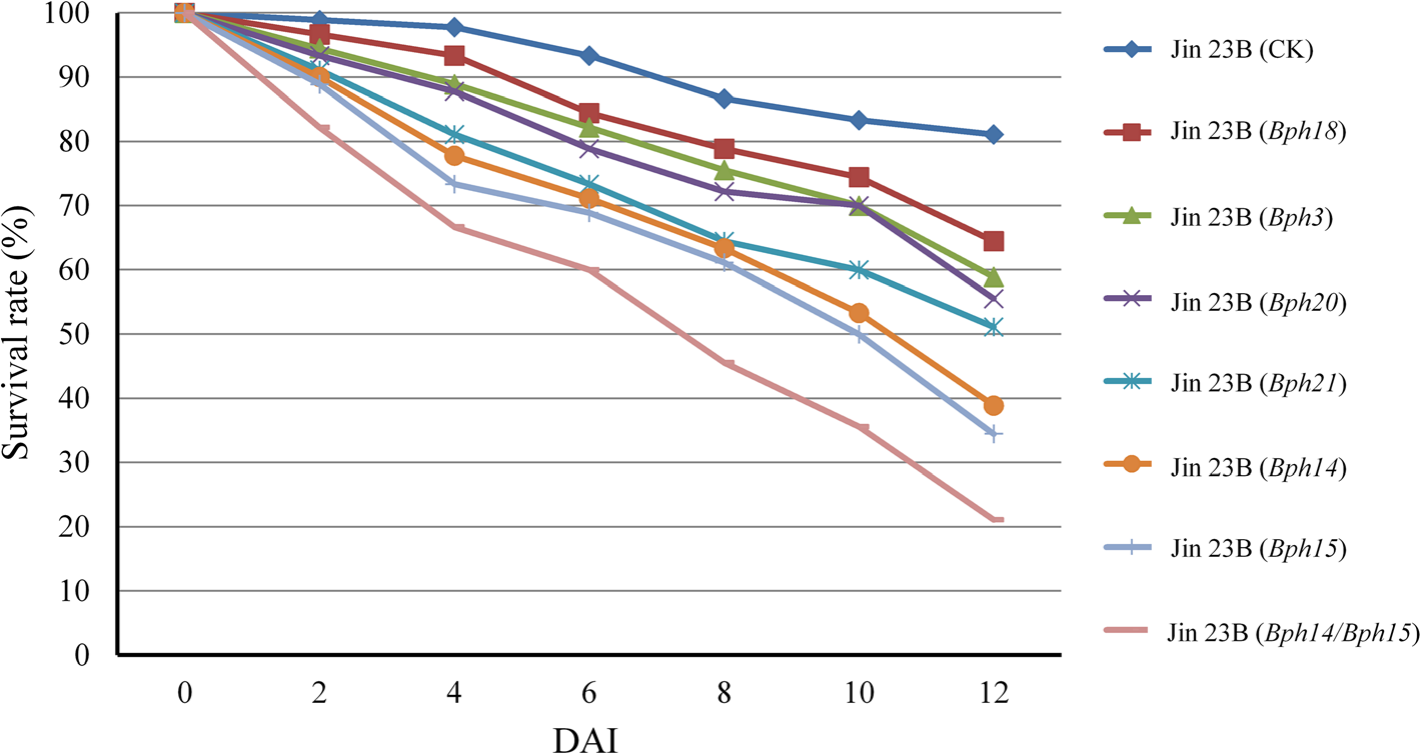
Survival rates of BPH on Jin 23B pyramiding line and introgression lines. The BPH survival rate was measured in every two days interval for 12 days. DAI: days after infestation

To know the influence of antibiosis in the BPH resistance *Bph3*, *Bph14*, *Bph15*, *Bph18*, *Bph20* and *Bph21*, the BPH survival rate was measured on the pyramided line and introgression lines in every two days interval for 12 days. As shown in Fig. 6, the average BPH survival rate on Jin 23B (14/15) was the lowest among all genotypes, which was 21.11% at 12 DAI. The average numbers of BPH surviving on Jin 23B (15), Jin 23B (14) and Jin 23B (21) decreased gradually and showed a significant difference in number compared with other genotypes at 8 DAI, and the average survival rates were 34.44%, 38.89% and 51.11%, respectively at 12 DAI. Comparatively, the average survival rates on Jin 23B (20), Jin 23B (3) and Jin 23B (18) were 55.56%, 58.89% and 64.45%, respectively at 12 DAI. For Jin 23B (none) and PTB33, the average survival rate was 81.11% and 42.53% at 12 DAI (Fig. 6).

Generally, these findings indicated that the BPHs probably had stronger effect on the antibiotic factors in the pyramiding line and introgression lines. And in the seven genotypes, the order of the gene effect on BPH survival at 8 DAI was: *Bph14*/*Bph15* > *Bph15* ≥ *Bph14* ≥ *Bph21* > *Bph20* ≥ *Bph3* ≥ *Bph18* > none.

### The maintainer ability of improved lines

In order to evaluate the fertility of the introgression lines as maintainers we assessed the pollen viabilities of hybrids with Jin 23A by I2-KI staining. Of the nine improved lines with *Bph3*, one line showed complete fertility (81.7%). Among the eight *Bph14* introgressed lines, two lines were completely fertile (94.0%) or partially fertile (53.0%). Among the eight *Bph15* introgressed lines, two lines showed completely fertile (70.7%) or partially sterile (15.0%). Among nine *Bph18* lines, two lines showed complete fertility (100% and 60.4%) and one line showed partially fertile (56.7%). Among ten *Bph21* lines, one line showed partial sterility (16.7%) and one line showed complete fertility (100%). Among eight *Bph14*/*Bph15* lines, one line showed partially fertile (48.3%). For *Bph20* introgressed lines, 6 lines were showed complete sterility (0%). The lines with fertile rate more than 0% in the F_1_ population were excluded, and all the improved lines were showed complete sterility (0%) in the BC_1_F_1_ population. Conclusion was that more than five lines of each genotype showed maintaining ability in the Jin 23B background.

### Agronomic traits of improved lines of Jin 23B

We compared the agronomic traits of one improved line from each of the 7 backcross populations with Jin 23B at Wuhan under stress-free conditions in 2013 (Table 2). Improved lines of Jin 23B (14), Jin 23B (18) and Jin 23B (14/15) were significantly lower in PH; and Jin 23B (20) had a significant increase in PN (Table 2). In addition, two improved lines, Jin 23B (3) and Jin 23B (20) had significantly decrease in NGP and SF; One improved line, Jin 23B (3), had significantly increase in GW. Furthermore, the yields of improved lines were found similar to the control (Jin 23B) under natural field conditions (Table 2). Generally, these results showed that two improved lines Jin 23B (15) and Jin 23B (21) had similar agronomic trait relative to the control maintaining the elite agronomic traits.

**Table 2:** Agronomic traits of improved Jin 23B lines with BPH resistance genes

Background	Heading date (days)	Plant height (cm)	No. of tillers per plant	No. of grains per panicle	Spikelet fertility (%)	1000-grain weight (g)	Single-plant yield (g)
Jin 23B (CK)	63.4 ± 1.1	91.8 ± 3.4	10.4 ± 2.2	94.0 ± 7.5	80.5 ± 5.8	24.2 ± 1.8	23.4 ± 4.5
Jin 23B (*Bph3*)	63.9 ± 1.5	94.1 ± 5.1	9.8 ± 2.7	81.9 ± 23.2**	64.1 ± 11.8**	26.5 ± 4.9**	21.1 ± 9.3
Jin 23B (*Bph14*)	62.9 ± 1.0	87.3 ± 4.3*	10.1 ± 2.8	89.0 ± 17.8	78.3 ± 4.5	24.4 ± 1.9	21.6 ± 5.4
Jin 23B (*Bph15*)	62.6 ± 0.8	88.9 ± 5.8	10.3 ± 2.9	98.5 ± 14.1	82.9 ± 5.2	23.1 ± 2.4	23.3 ± 7.0
Jin 23B (*Bph18*)	62.7 ± 1.0	87.3 ± 4.0*	10.5 ± 2.8	94.6 ± 18.5	78.4 ± 5.7	23.4 ± 1.8	23.0 ± 6.9
Jin 23B (*Bph20*)	64.2 ± 1.0	93.8 ± 5.4	13.0 ± 4.2*	83.8 ± 11.0**	71.1 ± 10.7**	24.7 ± 1.6	26.7 ± 8.9
Jin 23B (*Bph21*)	64.0 ± 1.1	94.3 ± 7.2	11.8 ± 3.9	89.3 ± 15.2	78.3 ± 7.3	23.5 ± 2.1	24.5 ± 8.9
Jin 23B (*Bph14/15*)	62.7 ± 0.9	88.6 ± 5.3*	9.6 ± 3.2	98.7 ± 20.8	81.1 ± 5.6	24.2 ± 1.7	22.7 ± 8.0

Significantly different from the performance of the improved lines with Jin 23B at * *P* < 0.05 and ** *P* < 0.01

## Discussion

Mapping and cloning of functional genes in rice have facilitated MAS of genes for resistance to biotic stress in rice genetic improvement in recent years. Marker-assisted selection combined with backcross breeding is a powerful method of coping with defects in otherwise very susceptible rice cultivars which had application in bacterial blight, sheath blight, blast, stem borer, and gall midge resistance (Huang et al. 1997; Katiyar et al. 2001; Datta et al. 2002; Maruthasalam et al. 2007; Koide et al. 2010; Jiang et al. 2012). For BPH resistance, recent studies have focused on incorporating BPH resistance genes such as *Bph14*, *Bph15*, and *Bph18* into the elite rice cultivars Minhui 63, 9311 and their hybrids (Hu et al. 2012, 2013).

In the present study, six dominant BPH-resistance genes, *Bph3*, *Bph14*, *Bph15*, *Bph18*, *Bph20* and *Bph21* were introgressed or pyramided into an important hybrid parent Jin 23B using MAS-based backcrossing, and improved lines containing single or two BPH-resistance genes were obtained. BPH bioassays showed that each of the six BPH resistance genes conferred different levels of resistance at both seedling and tillering stage of plants. The line with the combined *Bph14* and *Bph15* genes was more resistant than lines with the respective single genes and was the most resistant line overall. And the resistance level of single gene introgression line with *Bph15* was significant higher than that with *Bph3* or *Bph18* at seedling stage. Pyramiding line or introgression lines carrying gene *Bph14*/*Bph15*, *Bph15* or *Bph14* had a moderately resistant level at 25 DAI at tillering stage. And single introgression lines carrying *Bph20*, *Bph21*, *Bph3* or *Bph18* had a moderately susceptible level at 25 DAI at tillering stage. In honeydew weight and BPH survival rate, the pyramiding line with two genes had a significant difference from single introgression lines. And the single introgression lines had different among themselves (Figs. 3b, 6). The resistance levels of donor parent PTB33 at both seedling and tillering stages were significantly higher and the honeydew weight of PTB33 was significantly lower than Jin 23B (3), indicating that PTB33 may have more than one BPH resistance gene. In fact, Lakshminarayana and Khush (1976) reported that PTB33 carried two BPH resistance genes, namely *bph2* and *Bph3*. From the resistance level of Jin 23B (3), we hypothesis that Jin 23B (3) did not get the *bph2* gene from PTB33.

Gene effect of the BPH resistance conferred by *Bph3*, *Bph14*, *Bph15*, *Bph18*, *Bph20*, and *Bph21* was almost the same in honeydew weight, BPH survival rate, and the tests in seedlings stage. The pyramiding line carrying *Bph14* and *Bph15* has higher resistance level than the lines carrying single resistance gene. The results indicated an additive effect of pyramiding genes and showed that pyramiding two resistance genes have larger effects to BPH under Jin 23B background. In addition, single gene introgression line carrying *Bph15* had a largest effect than other single gene introgression lines. And single gene introgression line carrying *Bph18* had a lowest effect than other single gene introgression lines.

With the development of high-throughput and resolution genotyping platform in whole genome scale, whole genome SNP array-based background selection has become a feasible strategy with higher precision and efficiency for crop genetic improvement. The breeding chip RICE6K was employed in background examination of the BPH resistance improved lines. After three times backcross, all the improved lines had the segment of the target gene from donors. The genetic background recovery rate of Jin 23B (18), Jin 23B (21) and Jin 23B (14/15) were less than 90%, and the other segments from the donor parents may have had resistance QTL with positive effects on BPH resistance. Further backcrosses with Jin 23B would eliminate those segments. In addition, Jairin et al. (2007b) reported that IR71033–121-15 carried a QTL on short arm of chromosome 6. It would appear from the RICE6K, results that lines Jin 23B (20) and Jin 23B (21) did not get the chromosome 6 resistance QTL from the IR71033–121-15 donor.

Eight BPH-resistance genes (*Bph1*, *Bph2*, *Bph7*, *Bph9*, *Bph10*, *Bph18*, *Bph21*, *Bph26*) that are clustered on the long arm of chromosome 12, and they may be allelic. Among them *Bph26*, *Bph9* and *Bph18* have been cloned. Zhao et al. (2016) reported that *Bph9* shares 91.32% and 96.05% nucleotide sequence identity with *Bph26* and *Bph18*, respectively, and these allelotypes confer varying levels of resistance to different biotypes of BPH. The coding region of BPH1/9 shows a high level of diversity in rice germplasm (Zhao et al. 2016). In our study the allele *Bph18* and *Bph21* were introduced into Jin 23B and the resistance level conferred by *Bph21* was higher than *Bph18* at seedling stage.

Most of all, to pursue a durable and broad-spectrum resistance to BPH we should not only make use of gene pyramiding, but also exploit genetic diversity as an ecological approach to pest control, which can be highly effective over a large area and contribute to the sustainability of crop production (Zhu et al. 2000). It is more important that diversity represents insurance of buffering from failure of a single widely used gene. Because different BPH resistance genes are identified from different *indica* and wild rice accessions. Using of improved lines containing one or both BPH resistance genes planted in the different or same fields should restrict rapid increases in insect population and therefore reduce crop damage. Fortunately, *Bph14*, *Bph15* and *Bph18* have been pyramided into several rice hybrids in different genetic backgrounds, indicating that planting resistant pyramided hybrids in conjunction with conventional susceptible hybrids could effectively decreases the BPH population with the consequence of less serious BPH outbreaks, reduced costs of labor and pesticides, and increased rice production (Hu et al. 2011). The improved lines generated in this study can be used to develop multiple gene pyramided lines in the same genetic background for BPH control in hybrids.

Data from filed trails demonstrated that most of the required traits were well recovered in the improved lines (Table 2). The *Bph3* and *Bph20* introgression lines had a significantly lower NGP and SF relative to the control, but showed significantly increase in GW or PN. The yields of improved lines were similar to the control. Two improved lines Jin 23B (15) and Jin 23B (21) showed no difference in agronomic traits compared to Jin 23B. Jin 23A is an elite *indica* CMS line widely used in hybrid rice production in China. Through hybrid with Jin 23A, these BPH resistance gene can be transferred into Jin 23A, which can be used for development of BPH resistance hybrids in future. In addition, I2-KI stain of the F_1_ and BC_1_F_1_ hybrids show that the improved Jin 23B lines have good maintainer ability. These results show that these BPH resistance genes in Jin 23B have considerable commercial value for use in sustainable rice production.

From a global viewpoint, the increasingly severe occurrence of insects and diseases and indiscriminate pesticide is a challenge for sustainable rice production. A combination of approaches based on recent advances in genomic research has been formulated to address these challenges, with the long-term goal to develop rice cultivars referred to as Green Super Rice (Zhang 2007). Green Super Rice should possess resistance to multiple insect pests and diseases. In our previous work we have got the improved line Jin 23B (*Pi1*/*Pi2*/*D12*) containing three blast resistance gene (Jiang et al. 2012). This line can be hybridized with the present BPH resistance lines. The objective of the new improved lines is to address the problems of both blast and BPH.

## Conclusions

Overall, six dominant BPH-resistance genes *Bph3*, *Bph14*, *Bph15*, *Bph18*, *Bph20* and *Bph21* have an effect on BPH growth and development and antibiotic factors, resulting both seedling and tillering resistance. Bioassay test showed that the order of the gene effect being *Bph14*/*Bph15* > *Bph15* ≥ *Bph14* ≥ *Bph20* ≥ *Bph21* ≥ *Bph3* > *Bph18* > none at seedling stage of rice. Furthermore, field trial data demonstrated that yields of the improved lines were similar to the Jin 23B control under field conditions. These improved versions can be used in hybrid production in China.
